# Two strip flap technique for total lower lip reconstruction: case report

**DOI:** 10.3389/fmed.2025.1658394

**Published:** 2025-09-29

**Authors:** Wenli Qi, Xinyue Xiao, Jing Tong, Nengqiang Guo

**Affiliations:** ^1^Department of Plastic Surgery, Wuhan Hankou Hospital, Wuhan, China; ^2^Department of Plastic Surgery, Union Hospital, Tongji Medical College, Huazhong University of Science and Technology, Wuhan, China

**Keywords:** lower lip reconstruction, full lower lip repair, nasolabial flap, strip flap, oral cancer

## Abstract

**Background:**

Malignant tumor removal can cause extensive lower lip tissue loss, affecting speech, expression, and swallowing functions.

**Methods:**

We describe our experience with two cases using a modification of the nasolabial flap technique for total lower lip reconstruction. This approach employs two long strip-shaped flaps rotated to reconstruct the entire lower lip, which helps mitigate functional limitations caused by microstomia.

**Results:**

Two patients underwent successful reconstruction with normal lip function, no microstomia, and restored eating ability. Partial vermillion remained visible with good lip shape. No complications occurred during follow-up (12 months), with satisfactory oral competence and no drooling or speech impairment.

**Conclusion:**

This surgical method has a certain improvement in microstomia compared with the traditional surgery used for similar lower lip defects, while achieving satisfactory functional and aesthetic outcomes. The technique represents a preliminary technical note for total lower lip reconstruction with favorable outcomes in our limited series.

## 1 Introduction

Lower lip cancer is a common oral malignancy. Most lip cancers are categorized as squamous cell carcinomas, which originate from squamous cells found in the middle and outer layers of the skin (1). The risk factors for lip cancer include prolonged sun exposure and tobacco use (2, 3). The excision of lower lip cancer often results in defects in various areas of the lower lip tissue. Direct suturing will affect the morphology and even function of the lower lip, so it is necessary to repair and reconstruct through skin flap (4).

During the process of lower lip reconstruction, three crucial aspects require consideration: the anatomy of the lip, its subunits, and its functionality. Anatomically, the lip is composed of three distinct layers: skin, muscle, and mucosa. Situated as one of the focal points on the face, the lips occupy the lower-third of the facial region. The lip subunits extend from beneath the nose to the chin, with both the upper and lower lips comprising two components: the red and white portions, demarcated by the vermilion border. Further division can be made in the upper lip, creating three subunits separated by the middle of the manubrium and the nasolabial groove. This division included one medial subunit and two lateral subunits. Consequently, during the reconstruction process, preserving the lip red and subunit morphology becomes paramount (5). The lip plays a pivotal role in various functions including speech, facial expressions, swallowing, dental protection, and overall facial aesthetics. Therefore, any reconstruction method must aim to retain as much lip sphincter function as possible to fulfill these essential roles. Currently, the selection of techniques for addressing lower lip defects is often based on the size and nature of the defects. Smaller defects can frequently be managed through direct suturing or local flap transfers, whereas larger defects require a more tailored approach, such as utilizing local flaps, free flaps, or other suitable methods, contingent upon specific circumstances. Given the crucial role the lower lip plays in speech, facial expression, swallowing, and facial aesthetics, lower lip reconstruction must balance functional and aesthetic requirements (6).

A common challenge with many traditional techniques is the risk of microstomia, which significantly impacts patients’ quality of life by restricting oral function (7). In this article, we present our experience with a modified approach for total lower lip reconstruction using bilateral strip flaps. This surgical design is similar to von Bruns’ nasolabial flap, with the distinction that our method involves full-thickness incision of the skin at the nasolabial fold through to the oral mucosa, utilizing the buccal mucosa from inside the mouth to reconstruct the vermilion. We reported two cases with good postoperative results, and expect to provide a new technical reference for the repair of lower lip defects.

## 2 Report

### 2.1 Methods

#### 2.1.1 Flap design flow

**Figure 1:**
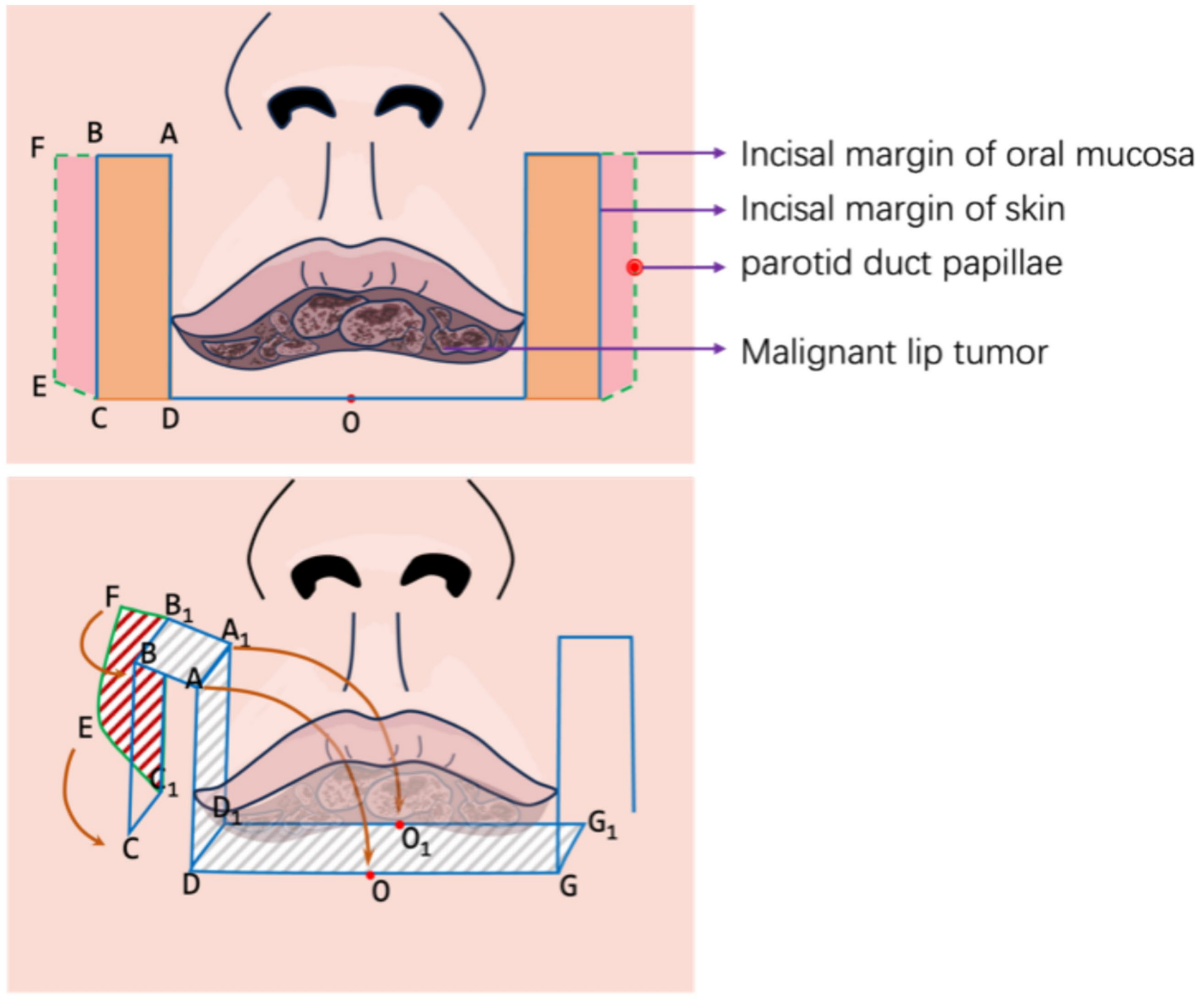
Flap design.

Our approach was performed under general anesthesia. The procedure consists of the following key steps (Figure 1).

**Figure 2:**
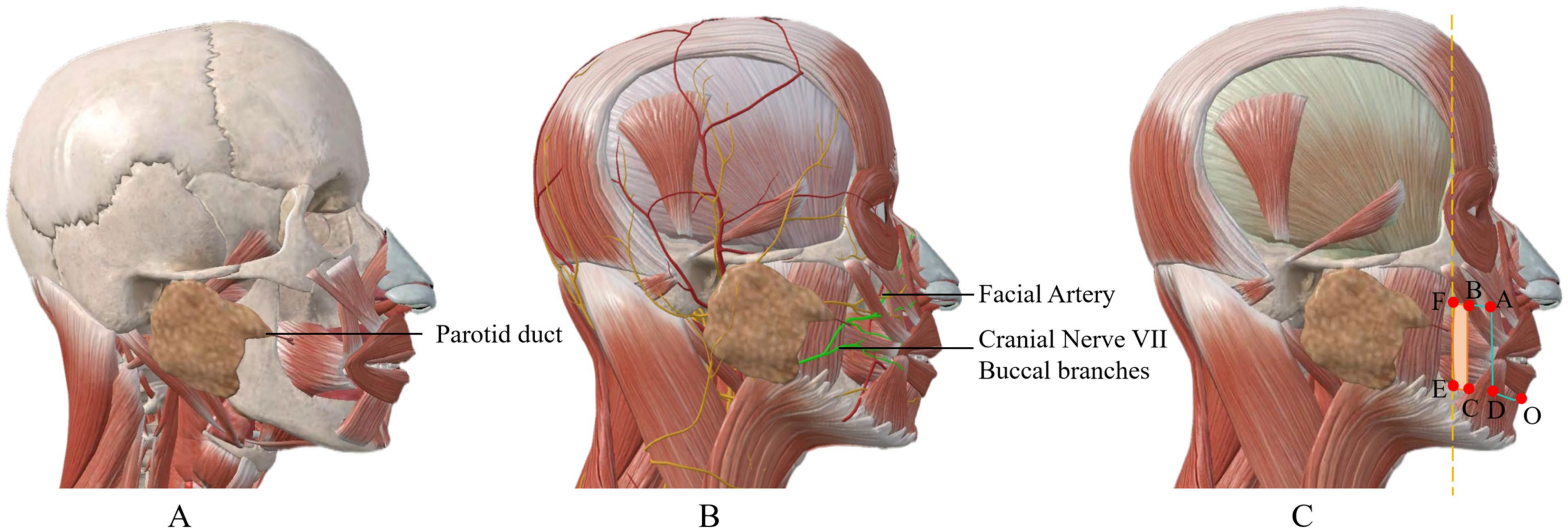
Overview of key anatomical structures related to the flap. **(A)** Shows the anatomical diagram of the parotid duct orifice, which is located at the outermost edge of the mucosa where the flap is harvested; **(B)** presents the anatomy of blood vessels and nerves; the buccal branches of the facial nerve and the branches of the facial artery pass through the location of the flap; **(C)** illustrates the anatomical positions of each marking point for the surgical incision of the flap.

1. Preoperative assessment and planning: thorough clinical evaluation including tumor size, depth of invasion, and lymph node status. Preoperative incisional biopsy was performed in both cases to confirm diagnosis.
2. Flap design: the contour of bilateral strip-shaped flaps was delineated using methylene blue. As shown in Figure 1, a long strip flap (ABCD) with width matching the excision area was designed along each oral commissure. The length of segment AD was preserved at 0.5 cm more than half of the defect length (AG) to ensure adequate tissue for flap apposition.
3. Tumor excision: full-thickness excision of the tumor with adequate margins (minimum 0.5 cm) was performed. Frozen section analysis was used to confirm clear margins intraoperatively.
4. Flap elevation: full-thickness incisions were made along AB, AD, and DO following the blue line markings. Critically, to reconstruct vermillio, the mucosal layer along BC was preserved and extended outward as much as possible, reaching to the parotid papilla. The green line in Figure 1 shows the incision margin of the mucous membrane. This full-thickness composite skin flap contains a named artery (facial artery branch) that must be preserved during dissection. As a full-thickness incised flap, some facial nerve branches may be damaged (Figure 2). After transfer, its anterior and posterior long edges are re-sutured. Regarding the method for identifying blood vessels, we localized them by palpating the vascular pulsations in the flap donor site and combining this with handheld ultrasound.
5. Vermillion reconstruction: following tumor excision, the reserved mucosal area was carefully separated, and the long edge of the mucosa (EF) was folded to cover the long edge of the flap (BC). Muscle layer: full-thickness aligned suturing. Using the slidability between muscle and mucosa, no separation of the two is done. Mucosa is sutured to skin without deliberate red lip reconstruction; part of buccal mucosa is sutured outward to substitute for some functions.
6. Flap rotation and suturing: after obtaining two strip flaps with CD serving as the pedicle, the flaps were rotated inward to cover the lower lip defect. The top ends of the flaps were apposed together to form the basic shape of the lower lip, followed by sequential suturing of the mucosa, muscle, and skin layers. For flap vermilion reconstruction, we use buccal mucosa reflection. However, limited by flap width, the vermilion cannot be fully restored to its original shape.
7. Postoperative care: patients received standard wound care and were followed closely for complications.

#### 2.1.2 Indication and contraindication

##### 2.1.2.1 Indications

Primarily for patients with narrow and long full-thickness lower lip defects. For such patients, the flap can better exert the advantage of saving the surrounding tissues.

##### 2.1.2.2 Contraindications

Not suitable for patients with large-area defects. The width of the flap cannot be made sufficiently wide, which may easily cause parotid gland dysfunction.

#### 2.1.3 Precautions

1. Prevention and management measures for skin flap hyperemia and ischemia.

The facial artery, running from near the oral commissure to the inner canthus, is preserved as the flap’s primary blood supply, largely preventing hyperemia and ischemia during surgery.

##### 2.1.3.1 For hyperemia

Relieve effectively by applying appropriate pressure with gauze postoperatively.

##### 2.1.3.2 For ischemia

Alleviate primarily by maintaining proper flap length-to-width ratio, avoiding narrow vascular pedicles, minimizing flap torsion, reducing pedicle compression, and preventing overly dense or deep sutures.

2. Measures to prevent scar contracture and vermilion retraction: The incidence of scar hyperplasia at the vermilion mucosa is relatively low. Additionally, the patients are elderly with relatively loose skin, leading to favorable outcomes for scar hyperplasia. In cases of scar hyperplasia, silicone gel massage can be used for scar treatment; if necessary, surgical adjustment may be performed.

## 3 Results

Two patients underwent successful reconstruction of the lower lip using our strip flap technique.

### 3.1 Case 1

**Figure 3:**
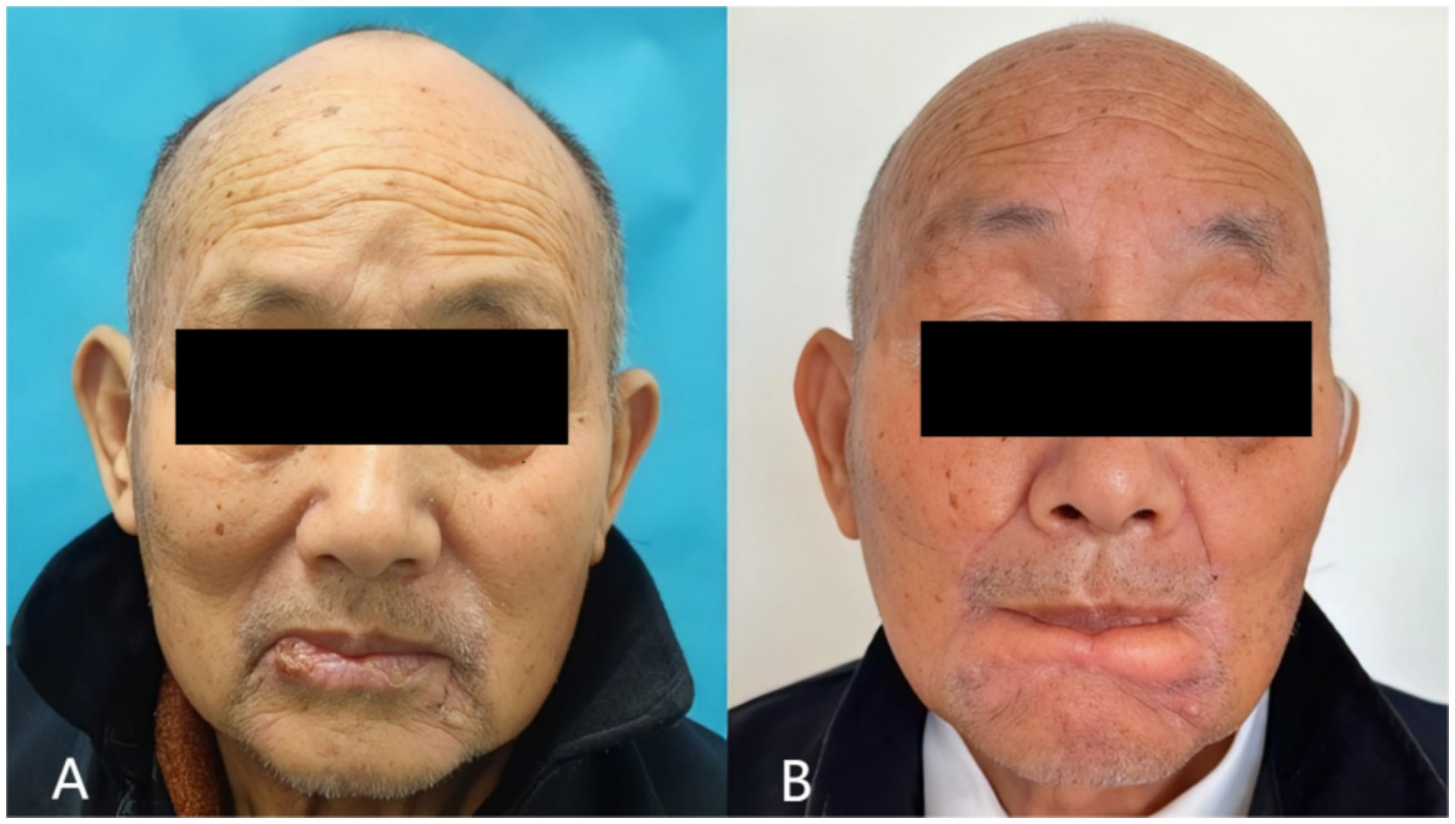
Preoperative **(A)** and postoperative **(B)** photographs of case 1.

An 80-year-old male patient presented with a painless ulcer on the lower lip persisting for several months. Physical examination revealed no cervical lymphadenopathy or other abnormalities. The comparison of preoperative and postoperative effects is shown in Figures 3A,B.

#### 3.1.1 Preoperative assessment

**Figure 4:**
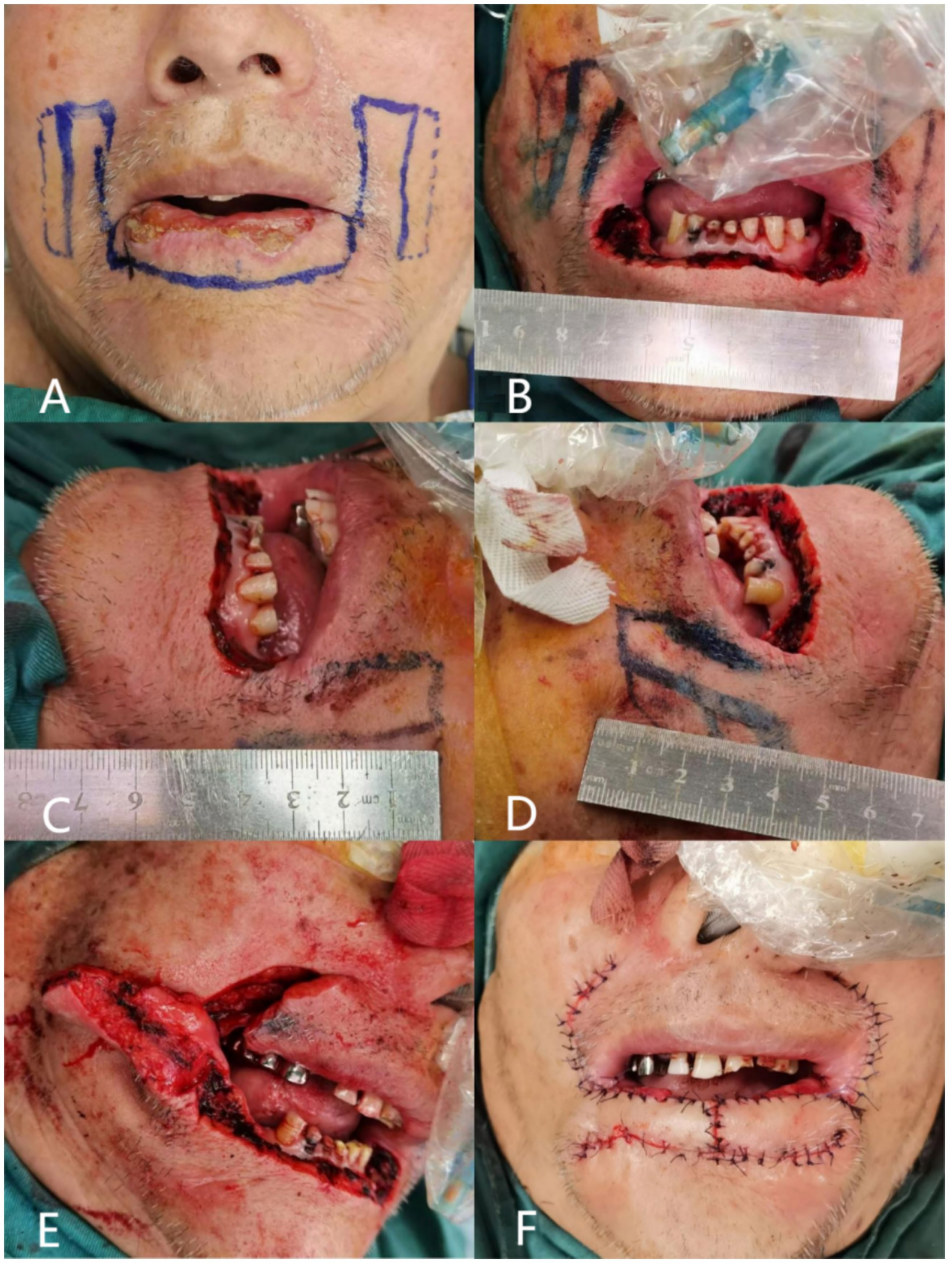
Surgical step of case 1. **(A)** Design of the flap margins; the full line marked is the skin margin, and the dotted line is the mucosal margin; **(B)** total excision of the malignant tumor of the lower lip, and the length of the lower lip defect is 7 cm; **(C)** length of the partial flap of the left lower lip, measured as 4 cm; **(D)** length of the partial flap of the right lower lip, measured as 4 cm; **(E)** separation of the partial flaps of the left and right sides, and the visible mucous membranes carried by the muscular layer; **(F)** suture after flap transfer.

Clinical examination showed two larger ulcerated surfaces measuring approximately 13 mm and 10 mm, with smaller ulcerated areas at the vermillion-mucosal junction (Figure 4A). Incisional biopsy confirmed moderately differentiated squamous cell carcinoma.

#### 3.1.2 Surgical intervention

A 7 cm × 1 cm full-thickness excision was performed with 0.5 cm margins around the tumor (Figure 4B). Intraoperative frozen section analysis confirmed clear margins. Two 4 cm long strip flaps were designed bilaterally (Figures 4C,D). The mucosal edge was extended approximately 0.5 cm to facilitate vermillion reconstruction (Figure 4E). The flaps were rotated medially and sutured as described in the methods section (Figure 4F).

#### 3.1.3 Postoperative outcome

The patient demonstrated excellent healing without wound complications. At 12-month follow-up, the patient maintained normal mouth opening and normal lip function, including the ability to pout (Figure 3B). No microstomia was observed, and the patient reported no dietary restrictions. Vermillion was visible when the mouth was opened, providing acceptable aesthetic results. Sensory assessment showed partial recovery of lip sensation at 12 months. No tumor recurrence was detected during the follow-up period.

### 3.2 Case 2

**Figure 5:**
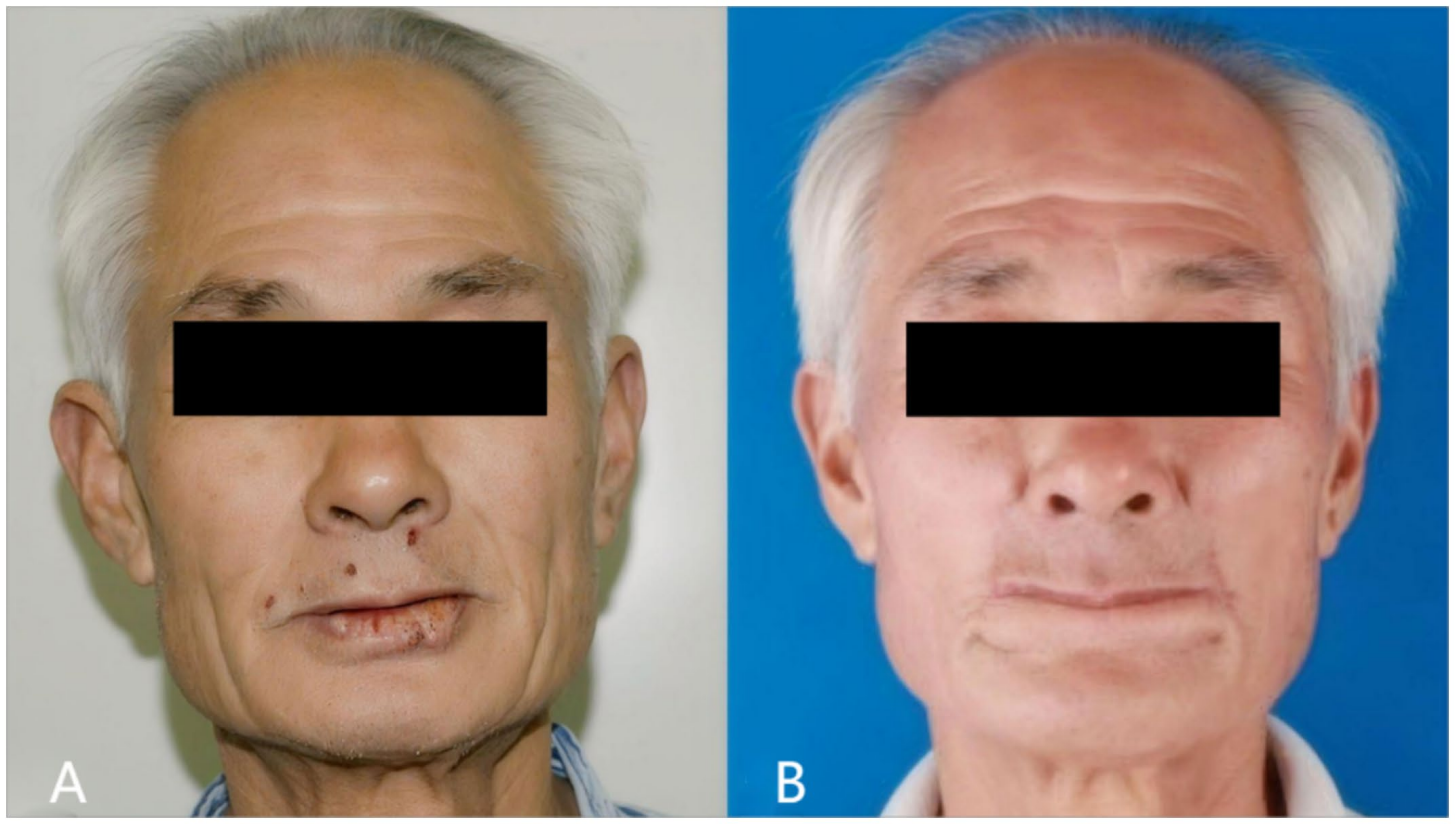
Preoperative **(A)** and postoperative **(B)** photographs of case 2.

A 78-year-old male patient was diagnosed with lower lip squamous cell carcinoma. The comparison of preoperative and postoperative effects is shown in Figures 5A,B.

**Figure 6:**
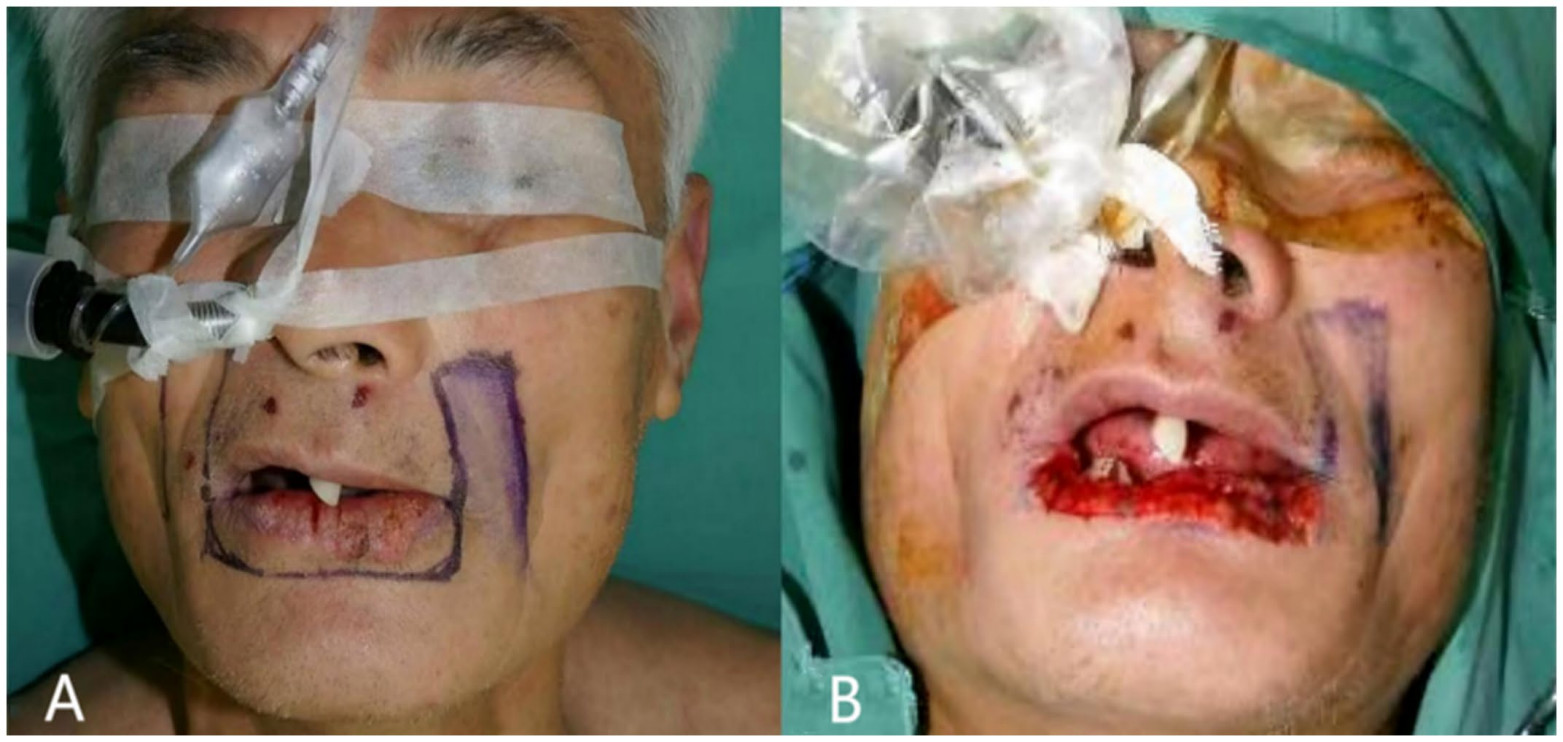
Surgical step of case 2.

Preoperative incisional biopsy confirmed well-differentiated squamous cell carcinoma. The patient underwent extensive local excision of the tumor and lower lip repair as described in Figures 6A,B.

The postoperative outcome is shown in Figure 5B. The patient achieved good cosmetic results, normal mouth opening, and normal eating. The patient reported no drooling or difficulty with oral continence. No tumor recurrence was observed during the 12-month follow-up period.

#### 3.2.1 Functional assessment

We used the Visual Analog Scale (VAS) to analyze patients’ oral function in detail (Table 1). Patients were tested on functions including mouth opening, oral mobility (chewing, cheek puffing, smiling) and speech ability, with a 0–10 scale (0 = worst, 10 = best). They rated each function independently on a paper strip, and average scores were calculated. Results showed the flap had little impact on mouth opening, oral mobility and speech ability, but greater impact on fine expressions like cheek puffing and smiling.

**Table 1:** Oral function VAS scores of 2 cases.

	Mouth opening (breathing & eating)	Oral mobility			Speech ability
		Chewing	Cheek puffing	Smiling	
Case 1	8.8	9.2	2.4	4.6	9.7
Case 2	9.1	9.5	1.0	4.3	9.8
Mean	8.95	9.35	1.7	4.45	9.75

**Figure 7:**
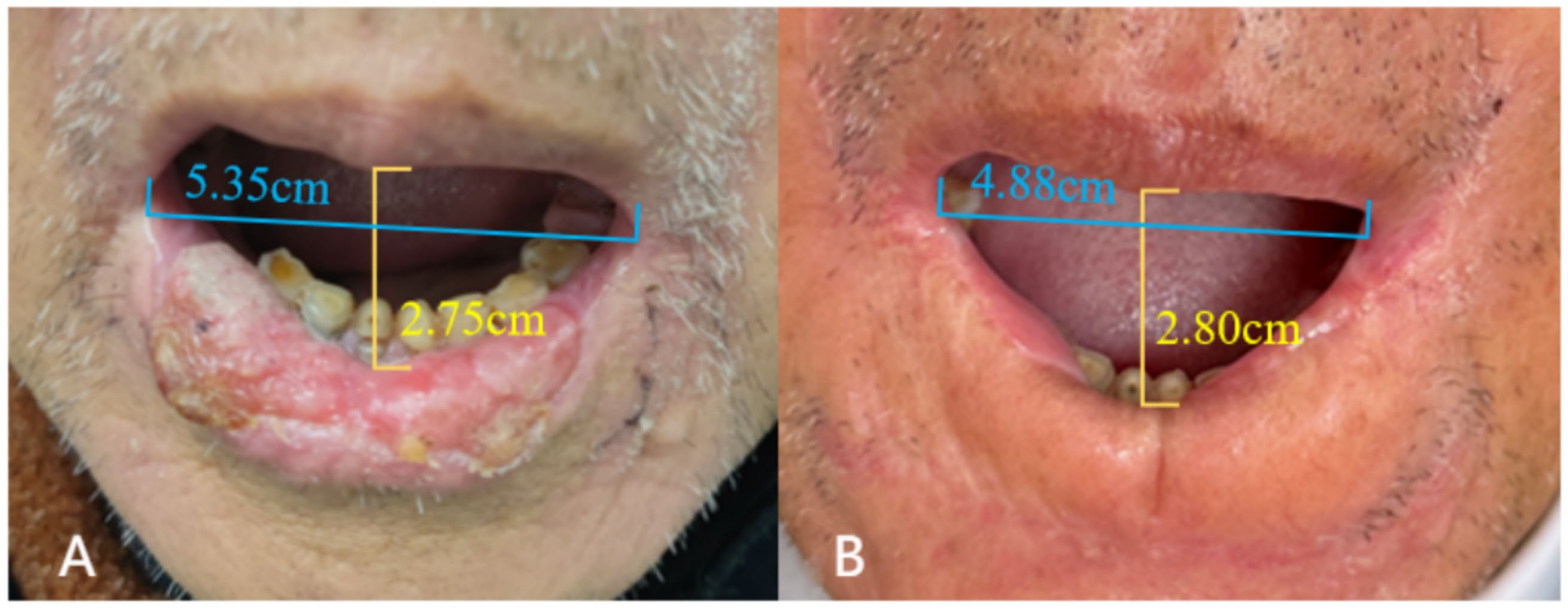
Mouth opening measurement of case 1: preoperative **(A)** vs. postoperative **(B)**.

For mouth opening, we measured the vertical distance between upper and lower lip margins and the distance between bilateral oral commissures (Figure 7). The vertical opening distance changed little (even slightly increased) vs. preoperatively, while the commissural distance decreased. Overall, mouth opening degree was slightly reduced, but minimally.

In terms of perioral sensation, the patient had no numbness in the perioral surgical area immediately post-op or at 1 year, and could perceive pain, temperature, touch, and pressure. However, long-term sensory follow-up was not done due to the patient’s age and long home-hospital distance.

#### 3.2.2 Cosmetic appearance evaluation

We evaluated two patients’ lip aesthetics via VAS, assessing parameters including symmetry, tissue thickness, vermilion retraction, hyperpigmentation, and cicatrization (Table 2). Using a 0–10 scale (0 = poorest, 10 = optimal), patients rated each parameter independently on a sheet, with mean scores calculated. Outcomes showed good flap performance in symmetry, hyperpigmentation and cicatricial hyperplasia, plus increased lower lip thickness and reduced vermilion definition.

**Table 2:** Appearance evaluation VAS scores of 2 cases.

	Symmetry	Tissue thickness	Vermilion retraction	Hyperpigmentation	Cicatrization
Case 1	8.8	6	5.0	8.5	8
Case 2	9.0	5.5	4.0	9	8.5
Mean	8.9	5.75	4.5	8.75	8.25

#### 3.2.3 Long-term outcomes

Both patients achieved favorable postoperative wound healing, with no occurrence of flap ischemia, congestion, or necrosis. During the follow-up visits, no other complications were observed in the patients at the current stage.

## 4 Discussion

Lower lip reconstruction presents a formidable challenge, as patients afflicted by this defect often endure severe dysfunction and disability. Various surgical techniques, including local tissue reconstruction and free tissue transfer, can be used to address this issue. Different surgical methods have been recommended for different defects. The choice of the surgical approach depends on the specific characteristics of the defect.

**Figure 8:**
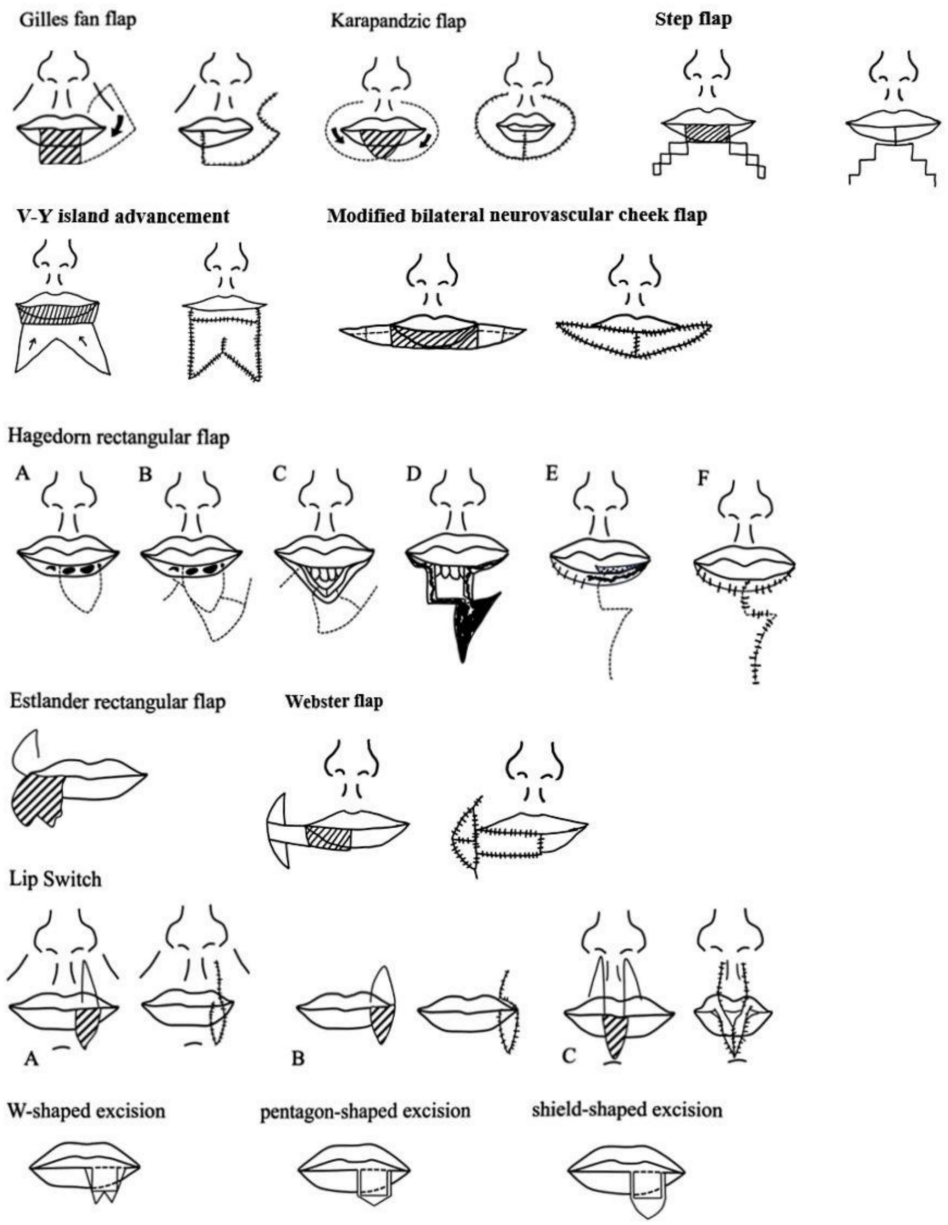
Summary of design of local skin flap for full layer repair of lower lip.

In this article, we conducted an extensive review of studies pertaining to lower lip reconstruction published on PUBMED between 1990 and 2024, as detailed in Table 3 and Figure 8. Our focus was to provide a comprehensive summary of the various approaches to full-lip repair.

**Table 3:** Summary table of the literature on full lower lip restorations.

	Typology	Literature sources	Type of defect	Type of flap
1	Local flap	Calhoun (1992) (13), Weerda et al. (1981) (15), and Ebrahimi et al. (2016) (16)	Macro defect	Gillies fan flap
2		Patel et al. (2024) (11) and Ebrahimi et al. (2016) (16)	Macro/large defect	Karapandzic flap
3		Chowchuen (2016) (17)	Large defect	Modified bilateral neurovascular cheek flap
4		Miyamoto et al. (2023) (18)	Large defect	V-Y island advancement
5		Russo et al. (2024) (19)	Moderate defect	Step flap
6		Brougham and Adams (2020) (20)	Moderate defect	Webster flap
7		Calhoun (1992) (13)	Moderate defect	Hagedorn rotational flap
8		Brabyn et al. (2018) (21)	Moderate defect	Estlander rotational flap
9		Calhoun (1992) (13)	Small defect	Lip switch technology
10		Calhoun (1992) (13) and Lu et al. (2011) (22)	Small defect	W-shaped excision
11		Huang and Arpey (1998) (23)	Small defect	Pentagonal resection
12		Huang and Arpey (1998) (23)	Small defect	Peltectomy (medicine)
13	Free flap	Gurunluoglu et al. (2012) (24)	Macro defect	Innervated thin femoral muscle flap
14		Tan et al. (2013) (25)	Macro defect	Free neural tendon fascia anterolateral femoral composite flap
15		Rahman et al. (2020) (26)	Macro defect	Free palmaris longus tendon flap
16		Khalid et al. (2024) (27)	Macro defect	Fibula free flap
17		Ansari et al. (2025) (28)	Macro defect	Forearm free flap

Local flaps offer a less invasive option and are suitable for addressing small-to medium-sized defects, as well as certain larger defects, contingent on factors such as the defect’s location, subunit-to-subunit association, and the patient’s priorities regarding future function and aesthetics. In cases where localized flaps might pose significant challenges, consideration of free flaps becomes relevant (8). A free flap can effectively cover the tissue defect and contribute to perioral capacity; however, it may fail to achieve the desired aesthetic outcomes and provide true functional muscle support for the lip.

When faced with a lower lip defect caused by extended resection of the malignant mass, two methods are usually considered: local flap transplantation and free flap (9). Local flap transplantation is a general method that utilizes blood circulation and structural features of the adjacent tissue to effectively restore the morphology and function of the lower lip. The advantages of this method are that it is simple, fast, and suitable for small defects. Nevertheless, if the defect is large, the free flap method is required. The free flap is a more complex procedure that requires the flap of other parts of the body to enter the lower lip defect. This method can be used to repair large defects and restore the blood supply and sensory function of the flap. However, this method is more difficult to perform and requires extensive surgical experience and techniques (10).

The Karapandzic flap is a fan-shaped flap that shares a basic shape and movement pattern with other flaps; however, it possesses its own dedicated blood and nerve supply (11, 12). The surgical procedure involved creating a skin incision parallel to the outer edge of the basiator, followed by careful dissection of the nerve and vascular pedicles. Subsequently, the muscle and mucosa around the vascular pedicle were divided, allowing complete rotation and suturing of the flap back into place. However, it can lead to reduced mouth opening, potentially interfering with normal lip closure, eating, speaking, and other daily activities. Additionally, it hinders the natural closure between the lower and upper lips, resulting in an aesthetically displeasing circular gap, which is often poorly accepted by patients (13). However, it is important to note that one notable advantage of this approach is the preservation of nerve function within the transferred tissue, leading to favorable functional outcomes (14).

The two long flaps designed here offer remarkable advantages compared to the Karapandzic flap (11). In terms of shape, these flaps retain the fundamental configuration of the lower lip strip while effectively concealing the scarring. Moreover, based on the patient’s prognostic photographs, we successfully preserved the natural redness of the lip to the greatest extent possible, maintaining the anatomical integrity of the lower lip, encompassing both the inner and outer skin. Functionally, the long strip flap discussed in this article mitigates the issue of microstomia, a concern associated with the karapandzic flap. This approach ensures that essential life functions, including speaking and eating, remain unaffected. Finally, our innovative flap can significantly reduce the difficulty of the surgical operation and shorten the operative time compared to the Karapandzic flap. Furthermore, the Karapandzic flap requires additional surgery at the donor site, which may increase the risks and complications of surgery. In contrast, our approach avoids this problem.

It is worth noting that there are limitations as well, as only a portion of the mucosa can be extracted owing to the constraints of the parotid papilla orifice, resulting in limited lip redness. Additionally, the morphology of the flap tip may require further refinement, which we intend to address in the third operation.

Lower lip reconstruction is quite challenging, and cases involving total or near-total lower lip reconstruction are particularly difficult. While a number of traditional surgical methods have been well-established for addressing different types of defects, the flap we designed specifically for narrow and elongated defects can, to a certain extent, provide a new preliminary approach for full-thickness repair of lower lip defects.

### 4.1 Optimized flap design

Unlike some surgical techniques described in previous literature, our method calculates the flap dimensions based on the size of the defect and adds an extra 0.5 cm to achieve tension-free closure.

### 4.2 Vermilion reconstruction

We create full-thickness labiobuccal composite tissue flaps and extend mucosal incisions—with transverse incisions specifically used on the inner side of the mouth—to maximize the acquisition of usable vermilion tissue. Vermilion tissue is crucial for both lip function and aesthetic appearance.

### 4.3 Preservation of oral aperture

The flap we adopted can maintain sufficient oral aperture and reduce the incidence of microstomia (a complication) to a certain extent, thereby better improving the patient’s prognosis.

## 5 Conclusion

Each flap has its own advantages and disadvantages, and appropriate surgical methods should be selected according to the size and specific condition of the lower lip defect. Patient age, physical condition, surgical needs, physician experience, and skill level are all determinants. Therefore, when selecting a flap, it must be comprehensively and carefully evaluated according to the specific situation of the patient to ensure that the patient achieves the best surgical results.

## Data Availability

The original contributions presented in the study are included in the article/supplementary material, further inquiries can be directed to the corresponding authors.
